# Novel **STMN2** Variant Linked to Amyotrophic Lateral Sclerosis Risk and Clinical Phenotype

**DOI:** 10.3389/fnagi.2021.658226

**Published:** 2021-03-26

**Authors:** Frances Theunissen, Ryan S. Anderton, Frank L. Mastaglia, Loren L. Flynn, Samantha J. Winter, Ian James, Richard Bedlack, Stuart Hodgetts, Sue Fletcher, Steve D. Wilton, Nigel G. Laing, Mandi MacShane, Merrilee Needham, Ann Saunders, Alan Mackay-Sim, Ze’ev Melamed, John Ravits, Don W. Cleveland, P. Anthony Akkari

**Affiliations:** ^1^Perron Institute for Neurological and Translational Science, Nedlands, WA, Australia; ^2^Centre for Molecular Medicine and Innovative Therapeutics, Murdoch University, Perth, WA, Australia; ^3^Centre for Neuromuscular and Neurological Disorders, University of Western Australia, Nedlands, WA, Australia; ^4^School of Health Sciences, Institute for Health Research, The University of Notre Dame Australia, Fremantle, WA, Australia; ^5^Institute for Immunology and Infectious Disease, Murdoch University, Perth, WA, Australia; ^6^Department of Neurology, Duke University, Durham, NC, United States; ^7^School of Human Sciences, University of Western Australia, Nedlands, WA, Australia; ^8^Centre for Medical Research, Harry Perkins Institute of Medical Research, The University of Western Australia, Perth, WA, Australia; ^9^Faculty of Medicine, The University of Notre Dame Australia, Fremantle, WA, Australia; ^10^Department of Neurology, Fiona Stanley Hospital, Murdoch, WA, Australia; ^11^Zinfandel Pharmaceuticals, Chapel Hill, NC, United States; ^12^Griffith Institute for Drug Discovery, Griffith University, Nathan, QLD, Australia; ^13^Ludwig Institute for Cancer Research, University of California, San Diego, La Jolla, CA, United States; ^14^Department of Cellular and Molecular Medicine, University of California, San Diego, La Jolla, CA, United States; ^15^Department of Neurology, Massachusetts General Hospital, Harvard Medical School, Boston, MA, United States; ^16^Department of Neurosciences, University of California, San Diego, La Jolla, CA, United States

**Keywords:** amyotrophic lateral sclerosis, genetic variant, genetic association studies, stathmin-2, genetic marker, structural variation, motor neuron disease

## Abstract

**Objective:**

There is a critical need to establish genetic markers that explain the complex phenotypes and pathogenicity of ALS. This study identified a polymorphism in the Stathmin-2 gene and investigated its association with sporadic ALS (sALS) disease risk, age-of onset and survival duration.

**Methods:**

The candidate CA repeat was systematically analyzed using PCR, Sanger sequencing and high throughput capillary separation for genotyping. Stathmin-2 expression was investigated using RT-PCR in patient olfactory neurosphere-derived (ONS) cells and RNA sequencing in laser-captured spinal motor neurons.

**Results:**

In a case-control analysis of a combined North American sALS cohort (*n* = 321) and population control group (*n* = 332), long/long CA genotypes were significantly associated with disease risk (*p* = 0.042), and most strongly when one allele was a 24 CA repeat (*p* = 0.0023). In addition, longer CA allele length was associated with earlier age-of-onset (*p* = 0.039), and shorter survival duration in bulbar-onset cases (*p* = 0.006). In an Australian longitudinal sALS cohort (*n* = 67), ALS functional rating scale scores were significantly lower in carriers of the long/long genotype (*p* = 0.034). Stathmin-2 mRNA expression was reduced in sporadic patient ONS cells. Additionally, sALS patients and controls exhibited variable expression of Stathmin-2 mRNA according to CA genotype in laser-captured spinal motor neurons.

**Conclusions:**

We report a novel non-coding CA repeat in Stathmin-2 which is associated with sALS disease risk and has disease modifying effects. The potential value of this variant as a disease marker and tool for cohort enrichment in clinical trials warrants further investigation.

## Introduction

Stathmin-2 (encoded by *STMN2*), has recently been reported as a potential contributor to the pathogenesis of ALS (Klim et al., 2019; Melamed et al., 2019). *STMN2*, a member of the stathmin family of proteins, encodes a phosphoprotein that has an important role in microtubule dynamics (Cook and Petrucelli, 2019). Microtubules are essential in maintaining the integrity of axonal signal transduction and cellular transport systems, as well as being important in neuronal growth and cell division (Kapitein and Hoogenraad, 2015). Core to the role of microtubules is their “dynamic instability,” namely the ability to switch between a state of rapid shrinkage and polymerization (Kapitein and Hoogenraad, 2015), which is largely mediated by microtubule associated proteins such as stathmin.

The Stathmin-2 protein promotes microtubule dynamics in axonal growth cones (Riederer et al., 1997; Morii et al., 2006), and is thought to play an important role in neurite outgrowth (Stein et al., 1988). Following nerve crush injury, Stathmin-2 is upregulated in the growth cones of regenerating axons (Shin et al., 2014), with mutant *STMN2* expression being associated with retraction of motor neurons from innervated neuromuscular junctions in *Drosophila* (Graf et al., 2011). Additionally, neurite growth is impacted by *STMN2* knockdown in human motor neurons, and these cells fail to exhibit extension compared to wild-type *STMN2* cell lines (Klim et al., 2019). Therefore, Stathmin-2 has been proposed as an axonal maintenance factor that, when depleted, can accelerate neurodegeneration, making this gene highly relevant in the context of ALS (Klim et al., 2019; Melamed et al., 2019).

Stathmin-2 is highly regulated by the RNA splicing factor TDP-43 (encoded by Transactive response DNA binding protein-43 kDa, *TARDBP*) that forms hallmark neuronal protein aggregates in both familial and sporadic ALS (sALS) (Nguyen et al., 2018), and knockdown of TDP-43 was recently shown to significantly reduce *STMN2* transcript levels (Klim et al., 2019; Melamed et al., 2019). TDP-43 plays a critical role in the repression of non-conserved cryptic exons, thus when TDP-43 aggregation and loss of function occurs, it facilitates the inclusion of a cryptic exon in the *STMN2* transcript (Ling et al., 2015; Melamed et al., 2019). Such conservation leads to the translation of an early stop codon resulting in a non-functional protein (Klim et al., 2019; Melamed et al., 2019). Reduced *STMN2* expression has been reported in spinal cord sections and human motor neurons of ALS patients, and has been attributed to this early polyadenylation mechanism (Klim et al., 2019; Melamed et al., 2019).

In the current study, the CA repeat variant of *STMN2* was initially identified *in silico* using a short structural variant evaluation algorithm (Saul et al., 2016), and was investigated using PCR, capillary separation and Sanger sequencing. A cross-sectional case-control study based on differential CA allele and genotype frequencies was performed in a combined North American sALS cohort, to determine if repeat length and allele/genotype frequencies are associated with disease risk, age of symptom onset, and survival duration. In addition, effects of CA genotypes on disease progression and cumulative survival were investigated in a smaller longitudinal follow-up cohort of Australian sALS patients. *STMN2* expression studies were carried out in olfactory neurosphere-derived (ONS) cells from sALS patients and controls, and in laser-captured postmortem spinal motor neurons, with accompanying RNA sequencing data (Krach et al., 2018).

## Materials and Methods

### Study Participants for Case-Control Studies

A combined cohort of 321 Caucasian North American sALS patients and 332 Caucasian North American healthy age-matched controls were used in the study. DNA and clinical information including age of onset, site of disease onset and survival duration, were collected from 152 sALS patients at the Duke ALS clinic in accordance with the Health Insurance Portability and Accountability Act (Pro00040665/323682). In addition, DNA samples from 169 sALS patients and 332 population control samples were obtained from the NINDS Repository, Coriell Institute for Medical Research (Camden, NJ, United States). Additionally, a longitudinal follow-up cohort of 67 Australian Caucasian sALS patients was obtained from the Sporadic ALS Australia (SALSA) project (HREC–2016-187, RGS1471, University of Western Australia RA/4/20/5308). DNA and clinical information, including age-of-onset, site of onset and ALSFRS scores at follow-up visits were collected for patients. All participants were diagnosed by board-certified neurologists and met the revised El Escorial World Federation of Neurology criteria for the diagnosis of ALS (Brooks, 1994).

### Polymerase Chain Reaction and Confirmation of Allele Length

To amplify the microsatellite, primers were designed to bind sequences on either side of the CA repeat with the forward primer located in exon 3 and the reverse primer located in intron 3 (sequences and cycling conditions are shown in Supplementary Table 1). Endpoint PCR reactions were prepared to a final volume of 10 μl, containing: 7.2 μl of dH_2_O (Baxter Healthcare, Old Toongabbie, NSW, Australia), 2 μl of MyFi reaction buffer (Bioline, Sydney, NSW, Australia), 0.05 μl of MyFi DNA polymerase (Bioline, Sydney, NSW, Australia), 0.375 μl of forward and reverse primer (Integrated DNA Technologies, Coralville, IA, United States) at 200 ng/μl, and 10 ng of DNA. PCR products were then fractionated on a 2% agarose (w/v) gel (Scientifix Pty Ltd., Clayton, VIC, Australia) in 1× TAE and stained with red safe nucleic acid stain (iNtRON Biotechnology, Scientifix Pty Ltd., Clayton, VIC, Australia) prior to imaging using the Bio-Rad ChemiDoc MP Imaging System. In heterozygous cases, individual amplicons were purified by band-stab, (Bjourson and Cooper, 1992) and Sanger sequenced by the Australian Genome Research Facility (AGRF) (Perth Australia). Analysis was conducted using Finch-TV software (version 1.5.0; Geospiza Inc.).

### High-Throughput Genotyping

Due to the variability of the repeat region, genotyping was performed via capillary separation using fluorescent end labeled primers, as a way to improve genotype resolution. PCR reactions were carried out using the above protocol except for the inclusion of a FAM-labeled forward primer. PCR products were sent for capillary separation at the AGRF (Perth Australia). Results were analyzed on peak scanner software (version 1.0; Thermo Fisher Scientific).

### Olfactory Neurosphere-Derived Cell Cultures and Immunocytochemistry

Olfactory mucosa biopsies were collected from four male sALS patients and four healthy, age-matched control participants. ONS cells (ONS cells) were generated from each biopsy according to our previously described protocols (Féron et al., 2013). All procedures were performed with written consent from the participants and with approval by the human ethics committee of Northern Sydney Local Health District (Sydney, Australia) and the Griffith University Human Ethics Committee (Queensland, Australia). The ONS cells were cultured in DMEM/F12 (1:1) containing 10% fetal bovine serum (v/v) and fixed using previously described methods (Gorecki et al., 2019). ONS cells were washed in PBS + 0.2% Triton X and blocked in 3% goat serum for 1 hr. Cells were incubated overnight at 4°C with Nestin (1:100 Thermo Fisher Scientific, Waltham, MA, United States, MA1-110), NeuN (1:500 Abcam, SYD, AU, ab177487) and β-tubulin (1:1000 Abcam, SYD, AU, ab18207) primary antibodies. Following washes, cells were incubated at room temperature for 1 h with secondary antibodies Alexa Fluor 488 (1:400, Bio-Rad, Hercules, CA, United States, ab150077) and Alexa Fluor 568 (1:400, Bio-Rad, Hercules, CA, United States, ab175701). Nuclei were stained with DAPI 0.5 μg/ml (Sigma-Aldrich, St. Louis, MO, United States) with final images taken on a Cytation 5 cell imager (BioTek, Winooski, VT, United States).

### RT-PCR

RNA was extracted using TRIzol (Thermo Fisher Scientific, Waltham, MA, United States) as per manufacturer’s instructions and resuspended in 30 μl of dH_2_O (Baxter Healthcare, Old Toongabbie, NSW, Australia). cDNA was synthesized using the SuperScript IV system (Thermo Fisher Scientific, Waltham, MA, United States) in 20 μl reactions as per manufacturer’s instructions, using 10 μl of template RNA with a total of 500 ng. Endpoint PCR reactions for *GAPDH* and *TARDBP* were prepared according to the MyFi enzyme protocol described above, *STMN2* reactions amplifying across exons 1–2 were prepared to a final volume of 10 μl, according to the hot start Phusion polymerase protocol (Thermo Fisher Scientific, Waltham, MA, United States) (primer sequences and cycling conditions, Supplementary Table 1). PCR products were fractionated on a 2% agarose (w/v) gel (Scientifix Pty Ltd., Clayton, VIC, Australia) and stained with red safe nucleic acid stain (iNtRON Biotechnology, Scientifix Pty Ltd., Clayton, VIC, Australia) prior to imaging using the Bio-Rad ChemiDoc MP Imaging System, followed by densitometry using image J software (National Institutes of Health, Bethesda, MD, United States).

### Human Postmortem Tissue

Human postmortem tissue collections were approved by the Institutional Review Board (Benaroya Research Institute, Seattle, WA, United States IRB# 10058 and University of California San Diego, San Diego, CA, United States IRB# 120056) and were obtained using a short postmortem interval acquisition protocol that followed HIPAA-compliant informed consent procedures. All sALS patients from whom tissues were obtained met the modified EI Escorial criteria for definite ALS (Brooks et al., 2000). Age-matched control nervous system tissues were obtained from hospice patients once life support was withdrawn. The average postmortem interval was 3.5 h for controls and 4.6 h for sALS patients. Detailed methods for tissue collection and pathological screening have been described previously (Krach et al., 2018). Laser-captured motor neurones were micro-dissected from lumbar spinal cord sections and RNA was collected and sent for RNA sequencing analysis as previously reported (Rabin et al., 2010; Krach et al., 2018). DNA was collected for *STMN2* CA genotyping.

### Statistics

Case-control binary genotype associations were assessed using a Pearson’s Chi-squared test, while case-control logistic regression was used to assess joint genotypic effects according to allele size categories (long and short) based on previous rationales (Roses et al., 2010; Mis et al., 2017; Sproviero et al., 2017). General linear models were used for the prediction of age of onset by genotype category. Within the SALSA longitudinal study, patient clinical characteristics were assessed over time using general linear mixed models (LMMs). Naïve LMMs were used to assess whether patient clinical characteristics were significantly associated with ALSFRS scores over time. Corrected LMMs were constructed to assess the impact of *STMN2* genotype group (L/L) on ALSFRS, independently of covariates. Variables identified as being statistically significant were included in the multivariable corrected LMMs. Residual plots were examined for all models and no violations were noted. Survival times were assessed using Kaplan-Meir analysis and Cox proportional hazards models. A *p* value below 0.05 was considered significant. Analyses were carried out in IBM SPSS Statistics version 25.0 (IBM Co., Armonk, NY, United States).

## Results

### Identification of a Polymorphic Structural Variant in *STMN2*

**FIGURE 1:**
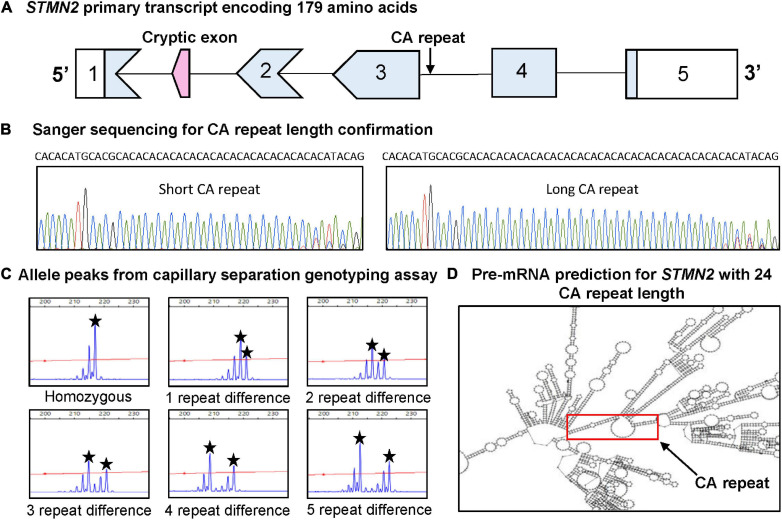
Identification and characterization of CA repeat polymorphism in *STMN2*. **(A)** Schematic of the *STMN2* primary transcript and the location of the CA repeat. **(B)** Sanger sequencing confirming size and variability of CA alleles. **(C)** Representation of *STMN2* CA alleles from capillary separation genotyping assay. The blue peaks depict the fluorescent signal intensity with the allele peaks to determine genotype indicated by the black star. **(D)** Predicted *in silico* pre-mRNA structure for *STMN2* reference sequence (NC_000008.11) generated from RNAfold Web server. The image depicts the predicted structure spanning from base pairs (79636317–79643806) with the red box indicating the portion of the pre-mRNA containing the variable CA repeat.

A structural variant evaluation algorithm (Saul et al., 2016), was used to search for polymorphic variants within *STMN2* that are likely to have significant biological effects contributing to disease risk. Structural variants were scored according to 24 different properties, previously described (Saul et al., 2016) and the candidate CA variant was selected from a short list for further investigation. The identified intronic CA repeat (Figure 1A) was investigated on public genomic databases NCBI and ensemble genome browser NC_000008.11 and 8:79641629-79641672, respectively, to see if the region had been previously resolved. Sanger sequencing revealed polymorphic CA alleles of different lengths (Figure 1B), suggesting that this candidate region is likely to be a highly informative genetic marker. Capillary fragment separation confirmed alleles ranged from 10 to 26 CA repeats (representative plots shown in Figure 1C). A subset of 7,490 base pairs across the *STMN2* gene (79636317–79643806) was mapped on RNAfold Web server (Institute for Theoretical Chemistry), to determine if the variable CA repeat altered secondary pre-mRNA structure. The predicted pre-mRNA structure was based upon the base pair sequence entered into the RNAfold Web server, therefore the structural prediction was the same for both cases and control with the same number of CA repeats. Interestingly, when CA repeat length was >18 CA, the repeat formed an open circular structure in the predicted pre-mRNA that was not present for those with <18 CA repeats (Figure 1D).

### Distribution of Alleles and Genotypes in North American sALS Cohorts

We next compared the distribution of the CA variant in the 152 Duke sALS cases, 169 Coriell sALS cases, and 332 healthy control cases (cohort demographics Table 1). The distribution of allele lengths ranged from 10 to 26 CA repeats in controls and sALS cases, and tended to fall in two groups: those with <19 CA repeats, classified as short alleles, and those ≥19 CA repeats that were classified as long alleles. Both patient groups consisted of individuals with self-reported Caucasian ethnicity and there were no significant differences in allele distributions between the Coriell and Duke cohorts (*p* > 0.8). Each distribution was compared to Webstr database and was similar to both GTEx (predominantly European self-reported ancestry) and 1000 genomes European allele distributions, providing confidence that the population in this study is reflective of a Caucasian population of European descent. The two patient groups were therefore combined to increase the sample size and power to detect genetic effects.

**TABLE 1:** Demographics of study participants used for case-control association.

	Duke cases (*n* = 152)	Coriell cases (*n* = 169)	Combined cohort (*n* = 321)	Controls (*n* = 332)
Males	82	92	174	167
Age (years)	57.3 (12.1)	56.8 (12.6)	57.0 (12.4)	55.6 (16.9)
Females	70	77	147	165
Age (years)	57.8 (11.9)	60.4 (13.4)	58.0 (12.6)	57.7 (13.9)
Age of onset	57.5 (12.0)	55.8 (13.7)	56.6 (12.9)	–
Disease duration (mths)	52.4 (38.1)	–	–	–

*Displayed as numbers of participants, means and standard deviations.*

### Long *STMN2* Alleles Are Associated With Disease Risk in North American sALS Cohorts

**FIGURE 2:**
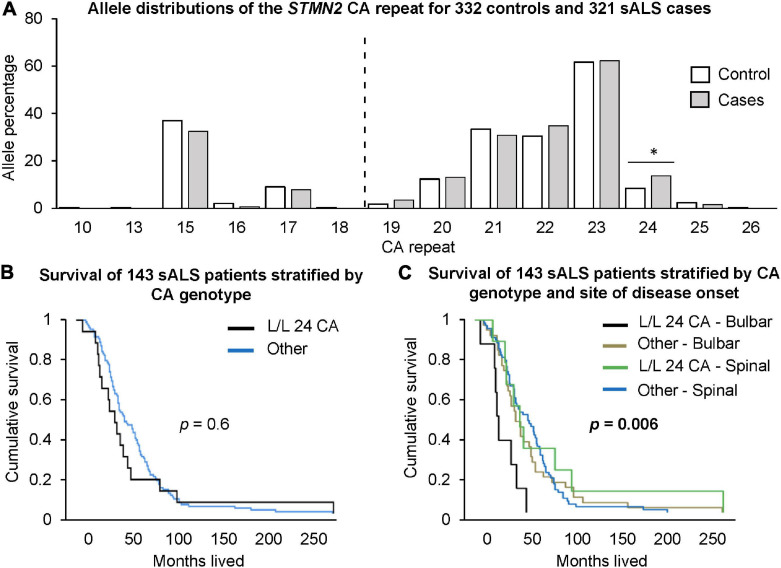
Investigation of *STMN2* CA genotypes and their association with sALS disease risk and survival. **(A)** Allele distributions of the *STMN2* CA repeat for 332 healthy controls and 321 sALS cases. The dotted line indicates the cut-off point between short and long CA alleles. Those <19 CA repeats are considered a short allele and those ≥19 CA repeats are considered a long allele. Significance is indicated by the *, *p* < 0.05. **(B)** Cumulative survival for 143 sALS patients from Duke University based on those that had L/L 24 CA genotypes compared to those without. **(C)** Cumulative survival based on site of disease onset (bulbar and spinal) and the presence of the L/L 24 CA genotypes.

There was a significantly higher frequency of genotypes with two long alleles (≥19 CA) in sALS cases compared to healthy controls (Table 2). In contrast, there was a significantly higher frequency of the long/short genotype in controls. At the individual allele level, only the presence of a 24 CA repeats was significantly associated with sALS cases, when it was part of a long/long genotype (*p* = 0.041, OR = 1.75, CI = 1.05–2.90, Figure 2A). To consider the impact of having two long alleles (≥19 CA) with the presence or absence of a 24 CA repeat, a case-control logistic regression was performed. In sALS cases there was a significantly higher frequency of those carrying two long alleles with at least one being the 24 CA repeat, whilst the frequency of those carrying two long alleles but without a 24 CA allele did not differ significantly between cases and controls. This effect was not abrogated by correction for age or gender.

**TABLE 2:** Distribution of CA genotypes in North American sALS and control cohorts.

Genotype	sALS	Controls	*p* value	OR	95% CI
Long/long (L/L)	203 (63.2%)	183 (55.1%)	0.042*	1.4	1.02–1.92
L/L (with a 24 CA)	37 (11.5%)	18 (5.4%)	0.0023**#	2.60	1.41–4.97
L/L (without 24 CA)	166 (51.7%)	165 (49.7%)	0.15#	1.27	0.92–1.76
Long/short (L/S)	105 (32.7%)	135 (40.7%)	0.035*	1.19	1.01–1.39
Short/short (S/S)	13 (4.0%)	14 (4.2%)	0.91	1.04	0.48–2.26

Bold values are statistically significant. Significance level is indicated by *. *p < 0.05, ** p ≤ 0.01. # p values from corrected model. OR, odds ratio; CI, confidence interval.

### Long *STMN2* Alleles Are Associated With Earlier Age-of-Onset in North American sALS Cohorts

To examine the effect of CA repeat length on age of disease onset, a generalized linear model was used. Within cases, carriage of at least one long allele (≥19 CA) was associated with a 7.5 years lower age at onset compared to other genotypes (*p* = 0.039, S/S = 63.85 years vs. other genotypes = 56.28 years; CI = 0.43–14.7).

### Long *STMN2* Alleles Are Associated With Reduced Survival in Bulbar-Onset Patients

End-point survival data was available for 143 of the 152 patients from the Duke University cohort. There was no significant difference in the survival times for those carrying two long alleles including 24 CA, and carriage of other genotypes (*p* = 0.6, Figure 2B). However, when categorized according to site of onset (bulbar vs. spinal), the bulbar cases carrying two long alleles including 24 CA had significantly shorter survival times than other bulbar cases (*p* = 0.0038, Hazard ratio [HR] = 3.2, CI = 1.45–7.0). There was no significant difference between survival times for bulbar cases not carrying the long alleles and spinal cases with or without the long alleles (*p* = 0.54). Relative to this latter combined group, bulbar cases carrying the long alleles, including the 24 CA repeat, had significantly shorter survival times (*p* = 0.006, HR = 3.4, CI = 1.6–7.1, Figure 2C). These results were not abrogated by adjustment for gender and age at disease onset.

### Long *STMN2* Alleles Are Associated With Increased Disease Severity in an Australian sALS Cohort

The relationship between *STMN2* CA alleles and disease severity and survival was also investigated in a cohort of 67 sALS patients from the SALSA project, who were followed up over periods of up to 25 months, with an average of 4 follow-up time points per patient (cohort demographics and clinical information are shown in Table 3). The frequency of CA genotypes in this cohort was: S/S = 3, S/L = 23, L/L = 41. With the limited number of participants in this cohort, sub-group analysis incorporating the 24 CA allele was not possible. To investigate the impact of *STMN2* CA genotypes on ALSFRS score over time, a generalized LMM was used which included significant predictors of change in ALSFRS, with age-at-onset, age at assessment, time between follow-up assessments, and site of onset as covariates. When accounting for repeated measures, the CA variant was significantly associated with mean ALSFRS scores over time (*p* = 0.034), with an overall reduction of 2.37 ALSFRS points in carriers of the L/L genotype (see Supplementary Table 2, estimated means from the model were L/L 28.98 vs. other genotypes 31.35). When the cumulative survival percentage was analyzed using a Cox proportional hazards model, comparing L/L genotypes to other genotypes, the L/L group showed a trend toward decreased survival duration compared to those with at least one short allele. Although not significant, the direction of the trend was consistent with our previous observations (see Supplementary Figure 1).

**TABLE 3:** Longitudinal clinical characteristics of follow-up Australian sALS cohort (SALSA) (*n* = 67).

Clinical characteristics		Mean (SD) or *n* (%)							
		Baseline (*n* = 67)	Visit 2 (*n* = 67)	Visit 3 (*n* = 51)	Visit 4 (*n* = 34)	Visit 5 (*n* = 24)	Visit 6 (*n* = 12)	Visit 7 (*n* = 3)	Visit 8 (*n* = 1)
Age at symptom onset (years)		61.92 (11.46)	–	–	–	–	–	–	–
Follow-up interval (mths)		–	6.34 (9.26)	11.39 (7.71)	15.45 (9.34)	19.97 (11.36)	24.21 (16.06)	60.94 (3.76)	25.9 (–)
Age at assessment (years)		65.31 (11.77)	65.83 (11.70)	64.85 (11.5)	64.75 (10.99)	63.20 (10.56)	64.70 (9.65)	60.94 (3.76)	59.31 (–)
Disease duration (years)		1.81 (2.42)	2.27 (2.48)	2.76 (2.64)	3.24 (2.74)	3.25 (2.31)	3.97 (2.89)	4.90 (1.51)	5.37 (–)
Sex	Male	38 (56.7%)	38 (56.7%)	31 (60.8%)	18 (52.9%)	15 (62.5%)	8 (66.7%)	3 (100%)	1 (100%)
	Female	29 (43.3%)	29 (43.3%)	20 (39.2%)	16 (47.1%)	9 (37.5%)	4 (33.3%)	–	–
Onset type	Spinal	49 (73.1%)	49 (73.1%)	36 (70.6%)	28 (82.4%)	20 (83.3%)	12 (100%)	3 (100%)	1 (100%)
	Bulbar	18 (26.9%)	18 (26.9%)	15 (29.4%)	6 (17.6%)	2 (16.7%)	–	–	–
ALSFRS		35.64 (8.4)	32.39 (8.38)	30.18 (8.19)	27.44 (9.98)	25.33 (9.02)	22.75 (9.90)	18.67 (6.66)	24 (–)

*Displayed as numbers of participants, means, and standard deviations. ALSFRS, amyotrophic lateral sclerosis functional rating scale.*

### *STMN2* Expression Is Reduced in sALS Olfactory Neurosphere-Derived Cells Compared to Controls

**FIGURE 3:**
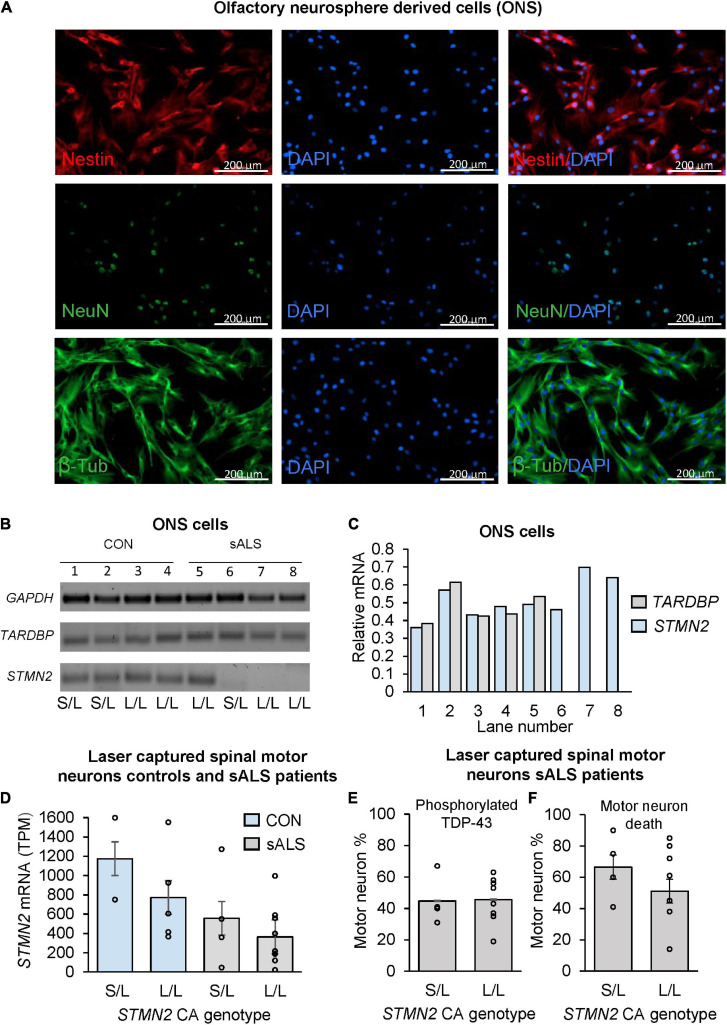
The effect of CA genotype on *STMN2* mRNA expression in olfactory neurosphere derived cell lines and laser captured spinal motor neurons. **(A)** Immunostaining of ONS cells with Nestin, NeuN, and β-tubulin. **(B)** Variable expression of *STMN2* in sALS ONS cell lines (lanes 5–8) compared to control ONS cell lines (lanes 1–4). **(C)** Relative densitometry of *TARDBP* and *STMN2* standardized to *GAPDH* expression showing variable expression of *STMN2* in sALS cell lines and no difference in *TARDBP*. **(D)** Trend for a stepwise decrease in *STMN2* mRNA expression between control and sALS cases according to *STMN2* CA genotype (S/L vs. L/L), error bars represent standard error of the mean. RNA sequencing data was analyzed by Krach et al. (2018) and *STMN2* expression between cases and controls previously reported (Melamed et al., 2019). **(E)** Percentage of motor neurons positive for phosphorylated TDP-43 during initial sample collection done by Krach et al. (2018), according to *STMN2* CA genotype. **(F)** Percentage of motor neuron death according to *STMN2* CA genotype based on data collected by Krach et al. (2018).

Previously, ONS cells have been used to model neurological disorders (Matigian et al., 2010; Mackay-Sim, 2012). Given the *STMN2* CA repeat was associated with sALS disease risk, age of onset and survival, we next asked whether the CA repeat explained variability in *STMN2* expression in control and sALS age matched ONS cells lines (see Supplementary Table 3). Immunostaining of primary ONS cells revealed expression of Nestin, NeuN and β-tubulin, demonstrating both progenitor/stem cells and mature neuronal characteristics (Figure 3A). A distinct difference in *STMN2* expression in ONS cell lines was evident between healthy control and sALS cases (Figures 3B,C). Within the control group, there was no observable difference in *STMN2* expression relative to CA repeat length; however, expression was detected in only one of the four sALS patient cell lines. No *STMN2* expression was detected for three of the patient cell lines, two of which had two long alleles including one patient with the risk allele. There was no observable difference in *TARDBP* expression between cases and controls (Figures 3B,C).

### Investigation of *STMN2* CA Genotypes and Expression in Postmortem Laser-Captured Spinal Motor Neurons

Tissue samples and RNA sequencing analyses were conducted on laser-captured motor neurons as previously reported (Krach et al., 2018). Based on this data, we genotyped DNA from these samples (see Supplementary Table 4), to examine if the CA genotype could help to explain the large amount of variability in *STMN2* within sALS cases that was not directly related to the presence of the cryptic exon, since all the patients were positive for the truncated *STMN2* transcript (Melamed et al., 2019). Once samples were stratified by the CA genotype, a distinct trend was observed in both patients and controls of decreasing *STMN2* expression between the S/L and L/L samples, however this did not reach statistical significance (Figure 3D).

We next examined if the relative TDP pathology was different between the CA genotypes of sALS patients (Figure 3E), when standardizing phosphorylated TDP-43 positive motor neurons to the number of morphologically intact motor neurons in laser-captured samples, and found that there was no difference between the percentages of motor neurons positive for phosphorylated TDP between the two genotypes. Additionally, there was no difference in the percentage of observed motor neuron death between the S/L and L/L genotyped patients (Figure 3F).

## Discussion

This is the first report of a structural variant within *STMN2* that is associated with sALS disease risk and also has disease-modifying effects. In a combined North American sALS cohort, the presence of at least one copy of the 24 CA repeat in genotypes consisting of two long alleles was found to be significantly associated with disease, thus confirming the hypothesis that allele length is a determinant of disease risk. Intuitively, if length is a driving factor, one could expect that carriage of short alleles would be protective, and our data supports this. In addition, the risk genotype group L/L (24 CA) was associated with reduced survival when accounting for initial site of disease onset and age at onset. Additionally, in a follow up longitudinal Australian sALS cohort, there was a significant reduction (2.3 points) in the mean ALSFRS score between the L/L and other genotype group. These results suggest that the CA variant may therefore be a potential marker to identify sub-groups of sALS patients that progress in a particular way, and could be a useful tool for cohort selection and enrichment in clinical trials.

*In silico* modeling of the identified CA repeat suggests that it has the potential to impact the binding of regulatory elements and alter pre-mRNA structure. This was of particular interest because it has previously been reported that hnRNP L (heterogeneous nuclear ribonucleoprotein L) can act as an expression enhancer and a regulator of splicing efficiency, with this function being directly related to CA repeat length of the target mRNA (Hui et al., 2003). Therefore, changes in CA repeat length could influence hnRNP L binding to the target pre-mRNA and potentially interfere with the binding of other regulatory elements at nearby sites that initiate splicing and regulate gene expression. To investigate effects on expression of *STMN2*, we measured *STMN2* mRNA in patient-derived ONS cells, taking into account respective CA genotypes. Whereas *STMN2* was detectable in all control cell lines independent of CA repeat length, expression was reduced in three of the four sALS cell lines investigated, in keeping with the previous observation that *STMN2* expression is reduced in ALS motor neurons (Klim et al., 2019; Melamed et al., 2019). Of particular note, two of the sALS cell lines with no detectable *STMN2* were found to carry longer versions of the CA repeat, including the 24 CA risk allele. Interestingly, the one sALS cell line that was positive for *STMN2* expression also shared a common 21 CA allele that was present in all four of the control cell lines. Despite being a long allele, this suggests that certain *STMN2* alleles may exhibit increased expression, and that the CA genotype may help to stratify differences in basal *STMN2* expression that are not accounted for by the TDP-43 cryptic exon mechanism (Klim et al., 2019; Melamed et al., 2019).

To further investigate this notion, we next examined *STMN2* CA genotypes in postmortem laser-captured motor neurons for which accompanying RNA sequencing data was available (Krach et al., 2018). It was reported previously that the average expression of *STMN2* was higher in controls compared to the sALS patients, with the cryptic exon being present in all of the sALS motor neurons (Melamed et al., 2019). However, this alone did not explain the variability of *STMN2* expression within the patient group, or the degree of cryptic exon present, and that the level of phosphorylated TDP-43 positive cells did not always correlate with the level of full length *STMN2* mRNA. Although not definitive, the present results exhibit a trend for a stepwise reduction in *STMN2* expression between the S/L and L/L genotypes, which was apparent both in control and sALS motor neurons, suggesting that the CA genotype may potentially reflect natural variability in the basal level of *STMN2* transcription. The observed trend appeared to be independent of the presence of phosphorylated TDP-43 in the collected motor neurons, suggesting that this variant may tag fluctuations in gene expression that cannot be identified purely by the level of cryptic exon expression.

The recently elucidated regulation of *STMN2* by the known RNA splicing factor TDP-43 suggests that *STMN2* may play a critical role in ALS pathophysiology (Klim et al., 2019; Melamed et al., 2019), particularly when TDP-43 pathology is present, making it a strong candidate for therapeutic development (Cook and Petrucelli, 2019). Since structural variants have the ability to affect gene regulation (Cleary and Ranum, 2014, 2017; Rose et al., 2016; Roses et al., 2016; Chiang et al., 2017), further expression studies of the different *STMN2* CA alleles are needed, particularly with the presence of the 24 CA repeat. If the 24 CA repeat results in reduced basal expression, this might further exacerbate disease progression in patients with the TDP-43 mediated cryptic exon mechanism (Ling et al., 2015), recently shown to play a significant role in maintaining axonal outgrowth (Melamed et al., 2019). Reduced *STMN2* expression has been reported not only in iPSC derived motor neurons in sALS and familial ALS cases with TDP-43 pathology, but also in motor cortex and spinal motor neurons of fALS and sALS patients with *C9orf72* repeat expansions, the most common inherited form of ALS (Melamed et al., 2019). Additionally, reduced *STMN2* expression has more recently been reported in the brain of patients with Parkinson’s disease (Wang et al., 2019), and frontotemporal dementia (Prudencio et al., 2020). Further supporting the finding we present here, it was also reported that levels of full length *STMN2* were not always correlated to phosphorylated TDP-43 burden in patients with frontotemporal dementia (Prudencio et al., 2020), suggesting that other factors outside the TDP-43/cryptic exon mechanism may contribute to regulation of *STMN2* expression.

Critical to understanding the implications of this data, is to assess the landscape of known genetic markers for sALS. Currently, there are only a few markers that explain increased disease risk, age-of-onset distributions, and variability in disease progression and survival (Blasco et al., 2011; Diekstra et al., 2012; ALSGEN, 2013; Lopez-Lopez et al., 2014; Fogh et al., 2016; Van Eijk et al., 2020; Yousefian-Jazi et al., 2020). Based on the data that we have generated, identification of this marker is the first step in fulfilling these requirements. As such, the *STMN2* multiallelic CA repeat is more informative than previously reported biallelic single nucleotide polymorphisms (Landers et al., 2009; Blasco et al., 2011; Diekstra et al., 2012; ALSGEN, 2013; Lopez-Lopez et al., 2014; Fogh et al., 2016; Theunissen et al., 2020), and may explain disease risk in a larger population of sALS patients. As a critical neuronal maintenance factor (Shin et al., 2014; Klim et al., 2019), attenuating *STMN2* expression may be a viable therapeutic approach to modify disease in a large proportion of both fALS and sALS patients, and reinforces its potential development for use as a prognostic marker or in clinical trials for ALS (Klim et al., 2019; Melamed et al., 2019) and other neurodegenerative diseases (Wang et al., 2019; Prudencio et al., 2020).

Importantly, we modeled the data using pre-determined long and short allele cut-off lengths to increase the power to detect a genetic effect, in a region that has a large number of genotypes. As such, alleles were grouped into length categories according to previous rationales (Roses et al., 2010; Mis et al., 2017; Sproviero et al., 2017). Although a limitation of the study, this decision was predicated on a clear distribution of alleles into two distinct length categories. However, one must consider that by grouping alleles, the frequency of long vs. short may change depending on selection of cut-off points, and could result in changes in individual allele frequencies. In order to accommodate this, we initially interpreted the data with caution until it was clear that the 24 CA allele (the longest allele at relatively high frequency) enhanced the effect initially detected for the long allele group.

## Conclusion

In conclusion, the *STMN2* 24 CA repeat, which was present in 11.5% of sALS cases in our combined cohort, and could potentially uncover disease risk in a significantly larger proportion of sporadic patients than the few existing genetic markers currently known for sALS. Moreover, the data that we present adds weight to the recently elucidated regulatory interactions between *STMN2* and TDP-43 in the pathogenesis of ALS. Such a discovery may work in parallel with the cryptic exon mechanism in regulation of *STMN2* expression and splicing efficiency. Our data suggests that the *STMN2* CA repeat may be a potential sporadic disease marker for ALS that can be used as a tool for cohort selection or to stratify variable patient response in clinical trials.

## Data Availability Statement

The raw data supporting the conclusions of this article will be made available by the authors, without undue reservation.

## Ethics Statement

The studies involving human participants were reviewed and approved by The University of Western Australia RA/4/20/5308, Duke University Health Insurance Portability and Accountability Act (Pro00040665/323682), Sporadic ALS Australia (SALSA) project (HREC–2016-187, RGS1471), and the Institutional Review Board (Benaroya Research Institute, Seattle, WA, United States IRB# 10058 and University of California San Diego, San Diego, CA, United States IRB# 120056). The patients/participants provided their written informed consent to participate in this study.

## Author Contributions

FT, RA, LF, and PA: concept design and study. RB: Duke University cohort sample collection and clinical information. NL, MM, and MN: SALSA patient recruitment, clinical data, and sample collection. AM-S: generation of the ONS cells. JR, ZM, and DC: generation of laser captured motor neurons and RNA sequencing data. FT: acquisition of data and initial draft of manuscript. FT, RA, LF, SJW, IJ, FM, and PA: analysis of data. All authors contributed to the manuscript editing and approved the manuscript.

## Conflict of Interest

FT, RA, LF, and PA hold the International patent application No. PCT/AU2020/051330 Discovery of informative structural variations within the STMN2 gene associated with sporadic Motor Neurone Disease. AS was employed by company Zinfandel Pharmaceuticals. The remaining authors declare that the research was conducted in the absence of any commercial or financial relationships that could be construed as a potential conflict of interest.
