# Supplementary material for: Novel STMN2 Variant Linked to Amyotrophic Lateral Sclerosis Risk and Clinical Phenotype
Source: Front Aging Neurosci. 2021 Mar 26;13:658226. doi: 10.3389/fnagi.2021.658226 (PMC8033025; doi:10.3389/fnagi.2021.658226)
Supplement: Supplementary file 1 [file Table_1.docx]

**SUPPLEMENTARY MATERIALS**

**Supplementary Table 1.** Primer sequences and cycling conditions

| Primer | Sequence | Cycling conditions | | | | |
| --- | --- | --- | --- | --- | --- | --- |
|  |  | **Hold** | **Denaturation** | **Annealing** | **Extension** | **Cycles** |
| CA_exon3 F | 5'-CCTGTCCCTGGAGGAGATCCA-3' | 95°C 4min30sec | 95°C  30sec | 56°C  30 sec | 72°C  1min | 35 |
| CA_intron3 R | 5'-CATGTTGGCATGGCACAGGTTC-3' |  |  |  |  |  |
| GAPDH_F | 5’-GAAGATGGTGATGGGATTTC-3’ | 95°C 4min30sec | 95°C  30sec | 57°C  30sec | 72°C  1min | 24 |
| GAPDH_R | 5’-GAAGGTGAAGGTCGGAGTC-3’ |  |  |  |  |  |
| TARDBP_F | 5’-CTTATGGTGCAGGTCAAGAAAGATCT3’ | 95°C 4min30sec | 95°C  30sec | 60°C  30sec | 72°C  1min | 35 |
| TARDBP_R | 5’-CGCTGTGACATTACTTTCACTTGTG-3’ |  |  |  |  |  |
| STMN2_exon1 F | 5’-CTTCTCTCTCGCTCTCTCCGC-3’ | 98°C  30sec | 98 °C  10sec | 57°C  30sec | 72°C  15sec | 40 |
| STMN2_exon2 R | 5’-CTTGTAGGCCATTGCTGTTTTAGCC-3’ |  |  |  |  |  |

**Supplementary Table 2.** General linear mixed model with predictors of ALSFRS score over time in Australian sALS patients

| Model Term | Coefficient | Std Error | t | *p* | 95% CI | |
| --- | --- | --- | --- | --- | --- | --- |
|  |  |  |  |  | **Lower** | **Upper** |
| Intercept | 34.30 | 3.46 | 9.91 | .000 | 27.49 | 41.11 |
| Age at symptom onset | .53 | .17 | 3.13 | .002** | .19 | .85 |
| Follow up time (mo) | -.18 | .06 | -3.13 | .002** | -.29 | -.07 |
| Age (yr) | -.59 | .16 | -3.69 | .000** | -.90 | -.27 |
| Spinal onset | 5.22 | 1.27 | 4.12 | .000** | 2.72 | 7.72 |
| Bulbar onset | 0^b^ | . | . | . | . | . |
| Other genotypes | 2.37 | 1.12 | 2.14 | **.034*** | .18 | 4.55 |
| L/L | 0^b^ | . | . | . | . | . |

**p* < 0.05, ** *p* = < 0.01, CI = confidence interval, 0^b^ = reference category

**
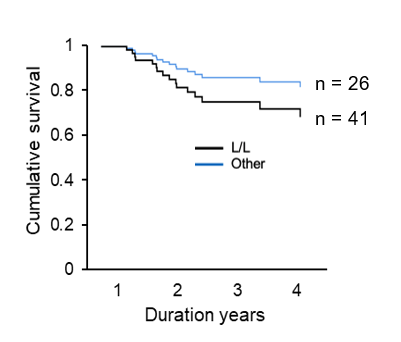
**

**Supplementary Figure 1.** Trend for decreased survival in the L/L genotype group compared to other *STMN2* CA genotypes in the Australian Caucasian longitudinal sALS cohort (*p* = 0.26).

**Supplementary Table 3.** Demographic information for ONS cell biopsies and associated *STMN2* CA genotype

| Status | Gel lane Fig (3B) | ID | Gender | Age of extraction | *STMN2* CA genotype |
| --- | --- | --- | --- | --- | --- |
| Control | 1 | 100140004 | Female | 47 | 15/21 |
|  | 2 | 100090004 | Female | 37 | 17/21 |
|  | 3 | 100140005 | Male | 49 | 21/23 |
|  | 4 | 100080013 | Male | 64 | 21/23 |
| sALS | 5 | C1500080001 | Male | 45 | 21/23 |
|  | 6 | C2500080002 | Male | 45 | 15/20 |
|  | 7 | 500070002 | Male | 37 | 23/23 |
|  | 8 | 500050001 | Male | - | 23/24 |

**Supplementary Table 4.** Demographic information for laser captured motor neurons, *STMN2* expression and CA genotype

| Control | ID | Gender | Age | Site of onset | Disease Course (yr) | Cause of death | PMI | *STMN2* (TPM) | *STMN2* CA genotype |
| --- | --- | --- | --- | --- | --- | --- | --- | --- | --- |
|  | 10 | Male | 78 | NA | NA | Sepsis/pulmonary hypertension | 2.5 | 367 | 22/23 |
|  | 39 | Male | 77 | NA | NA | Aortic dissection/MSF | 2 | 925 | 23/23 |
|  | 44 | Female | 80 | NA | NA | Liver failure | 5 | 415 | 23/23 |
|  | 65 | Male | 82 | NA | NA | NA | 4 | 1600 | 17/21 |
|  | 67 | Male | 77 | NA | NA | NA | 4 | 606 | 21/21 |
|  | 76 | Male | 68 | NA | NA | Sepsis, ARDS,ARF | 4 | 751 | 15/23 |
|  | 88 | Female | 78 | NA | NA | CVA,MSF | 3.8 | 1552 | 21/21 |
| sALS | 16 | Male | 61 | Arm | 2.5 | NA | 3.5 | 550 | 17/21 |
|  | 21 | Male | 84 | Respiratory and hand | 1.2 | NA | 2 | 996 | 21/23 |
|  | 27 | Male | 74 | Bulbar | 3.25 | NA | 4 | 359 | 17/21 |
|  | 34 | Female | 81 | Bulbar | 1 | NA | 3.5 | 22 | 20/21 |
|  | 48 | Male | 67 | Bulbar | 1.75 | NA | 6 | 217 | 21/23 |
|  | 60 | Female | 58 | Bulbar | 3 | NA | 3 | 558 | 21/22 |
|  | 62 | Male | 52 | Arm | 1.67 | NA | 6 | 590 | 23/23 |
|  | 63 | Male | 68 | Arm | 2.5 | NA | 5 | 399 | 21/22 |
|  | 79 | Male | 55 | Arm | NA | NA | 5 | 1274 | 15/24 |
|  | 82 | Male | 54 | Bulbar | 2.5 | NA | 8 | 192 | 21/23 |
|  | 84 | Female | 56 | Bulbar | 2 | NA | 4 | 192 | 21/22 |
|  | 85 | Female | 77 | Trunk | 2.3 | NA | NA | 120 | 22/23 |
|  | 89 | Male | 36 | Bulbar | 3 | NA | 5 | 187 | 15/21 |

*MSF multi system failure, ARDS acute respiratory distress syndrome, ARF acute renal failure, CVA cerebrovascular accident, PMI postmortem interval, TPM transcripts per million*
